# Procalcitonin-guided antibiotic therapy for pediatrics with infective disease: A updated meta-analyses and trial sequential analysis

**DOI:** 10.3389/fcimb.2022.915463

**Published:** 2022-09-21

**Authors:** Peng Li, JiaLe Liu, Junjun Liu

**Affiliations:** ^1^ The Key Laboratory of Geriatrics, Beijing Institute of Geriatrics, Institute of Geriatric Medicine, Chinese Academy of Medical Sciences, Beijing Hospital/National Center of Gerontology of National Health Commission, Beijing, China; ^2^ Department of Pediatric, Beijing Jingdou Children’s Hospital, Beijing, China; ^3^ Department of Oncology, Beijing Hospital, National Center of Gerontology, Institute of Geriatric Medicine, Chinese Academy of Medical Sciences, Beijing, China

**Keywords:** procalcitonin, pediatric, acute respiratory infection, antibiotic exposure, meta-analysis

## Abstract

**Objective:**

We aimed to evaluate the effect of procalcitonin (PCT) guided therapy on antibiotic exposure in pediatric patients with infectious disease.

**Methods:**

We performed an updated systematic review and meta-analysis of randomized controlled trials (RCTs) identified in systematic searches of MEDLINE, Embase, the Cochrane Database, Google Scholar, and SinoMed (through July 2021). The primary outcome was the length of the antibiotic therapy. Required information size (RIS) was calculated using trial sequential analysis (TSA).

**Results:**

Four RCTs with 1,313 patients with infectious disease were included. Overall, after a mean 22-day follow-up, PCT-guided antibiotic therapy was associated with a significantly shorter length of antibiotic therapy compared with the control group (WMD, −2.22 days; 95% CI, −3.41 to −1.03; P <0.001) and a decreased rate of antibiotic adverse events (RR, 0.25; 95% CI, 0.11–0.58; P <0.001). However, the length of hospital stay (WMD, −0.39 days; 95% CI, −0.84 to 0.07; P = 0.094), rates of antibiotic prescription (RR, 1.10; 95% CI, 0.97–1.25; P = 0.122), hospital readmission (RR, 1.03; 95% CI, 0.92–1.16; P = 0.613) and mortality (RR, 0.73; 95% CI, 0.17–3.19; P = 0.674) were comparable between the PCT-guided antibiotic and control groups. TSA showed that the RIS was 2,340, indicating a statistically significantly shorter length of antibiotic therapy between PCT-guided antibiotic and control groups (P <0.05).

**Conclusions:**

PCT-guided management seems to be able to decrease antibiotic exposure in patients with infectious disease. However, much larger prospective clinical studies are warranted to confirm these findings.

## Introduction

Procalcitonin (procalcitonin, PCT) is a new inflammatory indicator discovered by Assicot et al. in 1993. PCT is a more specific infection-related biological marker index for diagnosing bacterial infection than CRP (Wacker et al., 2013). In recent years, it has been recognized that PCT is a good indicator of the inflammatory response caused by bacterial infection. It is a precursor of calcitonin (CT) with no hormone activity. Its half-life is 25–30 h, and it has good stability *in vitro* and *in vivo*. It is not affected by hormone levels in the body. Under physiological conditions, thyroid C cells are the main cellular source of PCT (Simon et al., 2004). Under pathological conditions, the site of PCT production in the body is mainly located in the liver. Other neuroendocrine cells such as peripheral blood mononuclear cells, spleen, lung, or small intestine are also important sites for PCT production. It can hardly be detected in the serum of healthy people. In patients with systemic bacterial infection (especially sepsis and Gram-negative bacterial infection), the serum PCT level increases early, rapidly, and lasts for a long time, whereas in viral infection, in patients with autoimmune diseases, and in patients with localized infections, PCT levels are maintained in the normal range or slightly elevated (Pontrelli et al., 2017). PCT levels also increase significantly with the increase in the severity of infectious diseases. The serum PCT level increases 3 to 6 h after the onset of a bacterial infection, reaches a peak at 8 to 24 h, and has a half-life of 22 to 35 h. Therefore, levels of PCT can be used as good indicators for the early diagnosis of systemic bacterial infection and sepsis (Lee et al., 2020).

In addition to early, timely, and accurate diagnosis of systemic bacterial infection in children, active control of infection and correct selection of antibiotics are the keys to improving the success rate of treatment. In routine clinical practice, when the exact pathogenic bacteria and drug susceptibility results are unknown, the antibiotic therapy regimen is often empirical. Many conditions, such as the local and hospital microbiological data and antibiotic susceptibility, the severity of the disease of the child, the antibiotic susceptibility of common pathogens, and the risk of drug-resistant bacterial infections (Pletz et al., 2020). Initially, the broadest-spectrum antibiotics or combined antibiotics are often selected to strive to cover all possible pathogenic bacteria quickly to control the infection. When the bacterial culture and drug susceptibility results are available, narrow-spectrum antibiotics can be used in a targeted manner (Modi and Kovacs, 2020). However, empirical antibiotic therapy often has the problem of blind application or abuse of antibiotics, which not only affects the therapeutic efficacy, increases the economic burden, but also promotes the production of drug-resistant bacteria (Barreiro and Barredo, 2021).

Dynamic monitoring of PCT levels can be used to guide the rational use of antibiotics (Pletz et al., 2020). A large number of randomized controlled studies (RCT) have confirmed that PCT levels could be used to guide the initiation and/or stop of antibiotic therapy in community-acquired pneumonia (CAP), acute exacerbation of chronic obstructive pulmonary disease, acute bronchitis, and sepsis (Picart et al., 2016; Carpenter, 2018). Furthermore, during the COVID-19 pandemic, PCT testing was widely introduced at hospitals. However, the efficacy of PCT-guided treatment during the COVID-19 pandemic should be confirmed, though several studies indicated the potential to help COVID-19 patients with bacterial co-infection (Llewelyn et al., 2022; Wolfisberg et al., 2022). It can significantly reduce antibiotic exposure without affecting treatment outcomes. A 2017 published Cochrane systematic review analyzed data on 6,708 patients with acute respiratory infection from 26 RCTs and showed that the PCT-guided group had lower 30-day mortality (8.6% *vs*. 10%, P = 0.037), a 2.4-day reduction in antibiotic exposure time (5.7 days *vs*. 8.1 days, P <0.001), and antibiotic-related adverse events (16.3% *vs*. 22.1%, P <0.001) (Schuetz et al., 2017). Therefore, according to the level of PCT, a revised antibiotic therapy regimen for patients with suspected or confirmed bacterial infection can help reduce the exposure time to antibiotics.

However, no RCT published after 2016 demonstrated that PCT-guided management was associated with decreased antibiotic exposure compared with empirical care, which might be associated with the recent progress in antibiotic stewardship or access to infectious disease in the empirical care group (van der Does et al., 2018; Chomba et al., 2020). Meanwhile, the vast majority of RCTs supporting PCT-guided antibiotic therapy have been performed in adult patients, and the role of PCT-guided antibiotic therapy in pediatrics with infection is still very limited (Mathioudakis et al., 2017; Schuetz et al., 2018).

Therefore, as the evidence gathered has recently increased, we have performed a meta-analysis and a trial sequential analysis (TSA) to evaluate the effect of PCT-guided therapy on antibiotic exposure in pediatrics with infectious disease.

## Methods

### Data sources and search strategies

We searched MEDLINE (source, PubMed from 2005 to July 2021), Embase (2005 to July 2021), the Cochrane Controlled Clinical Trials Register Database (to July 2021), Google Scholar (to July 2021), SinoMed (to July 2021), and the ClinicalTrials.gov website (to July 2021) using the terms “infection,” “pneumonia,” “sepsis,” “procalcitonin,” “pediatric,” “children,” “length of antibiotic therapy,” and “randomized trial.” Manual reference checking of the bibliographies of all relevant articles was performed. No restrictions were applied. The review was registered at https://inplasy.com/.

### Study selection

We first conducted an initial screening of titles and abstracts; the second evaluation was based on a full-text review. Trials were considered eligible if they met these criteria: 1) pediatric patients with infection (pneumonia, low respiratory tract infection, sepsis); 2) pediatrics in the PCT group were given PCT-guided antibiotic therapy regimen; 3) pediatrics in the control group were treated with empirical antibiotic regimen; 4) primary outcome of interest was the length of antibiotic therapy; and 5) the study was an RCT. Exclusion criteria were (1) neonate, adult patients; (2) complicated with immune deficiency (malignancy, bone marrow transplantation, solid organ transplant), or infection requiring prolonged antibiotic therapy (endocarditis, mediastinitis, osteomyelitis); (3) single-arm study; (4) without primary outcome; (5) cohort study, retrospective study, animal study, case report, or review; and (6) duplicated data.

### Data extraction

Two reviewers extracted data concerning patient characteristics, the PCT-guided and empirical antibiotic therapy regimens used, study quality, and clinical outcomes using a standard data-collection form. Disagreements are resolved by discussion.

The primary outcome was the length of the antibiotic therapy. Secondary outcomes were the length of hospital stay, rates of antibiotic prescription, hospital readmission, mortality, and antibiotic adverse events.

### Quality assessment

The Preferred Reporting Items for Systemic Reviews and Meta-Analyses (PRISMA) statement (Moher et al., 2009) was followed. Two reviewers assessed the quality of the selected trials. Components used for quality assessment were means of generation of random sequence, allocation concealment, blinding of outcome assessment, and selective outcome reporting.

### Data synthesis and analysis

Results were analyzed quantitatively with STATA 14.0 software (Stata Corp, California, USA) using the fixed-effects model (DerSimonian and Laird, 1986). We calculated the pooled relative risk (RR) for dichotomous outcomes and the weighted mean difference (WMD) for continuous data with 95% confidence intervals (CI).

Heterogeneity was examined by the I^2^ statistic and the chi-square test. A value of I^2^ >50% was considered a substantial level of heterogeneity (DerSimonian and Laird, 1986). Once heterogeneity was noted, between-study sources of heterogeneity were investigated using subgroup analysis by stratifying original estimates according to study characteristics. Sensitivity analyses were conducted to determine the influence of individual trials on the overall pooled results. All analyses were performed according to the intention-to-treat principle. Statistical significance was set at 0.05 for the Z-test for RR.

### Trial sequential analysis

In the meta-analyses, trial sequential analysis (TSA) was used to reduce the risk of reaching a false-negative conclusion (Wetterslev et al., 2017). When the cumulative Z-curve crossed the trial sequential monitoring boundary or entered the futility area, a sufficient level of evidence for the anticipated intervention effect was reached, and no further trials were required. If the Z-curve did not cross any boundary and the required information size (RIS) had not been reached, the evidence to conclude that it was insufficient was more trials needed to confirm the results. In this TSA for the length of antibiotic therapy, we estimated the RIS based on a mean difference reduction of −2.0 days. The type I error (α) = 0.05 (two-sided) and power (1 − β) = 0.80. The variance was 20.0. The I^2^ value was 90%. TSA was conducted using TSA Version 0.9.5.10 Beta (www.ctu.dk/tsa).

## Results

### Search results

**Figure 1:**
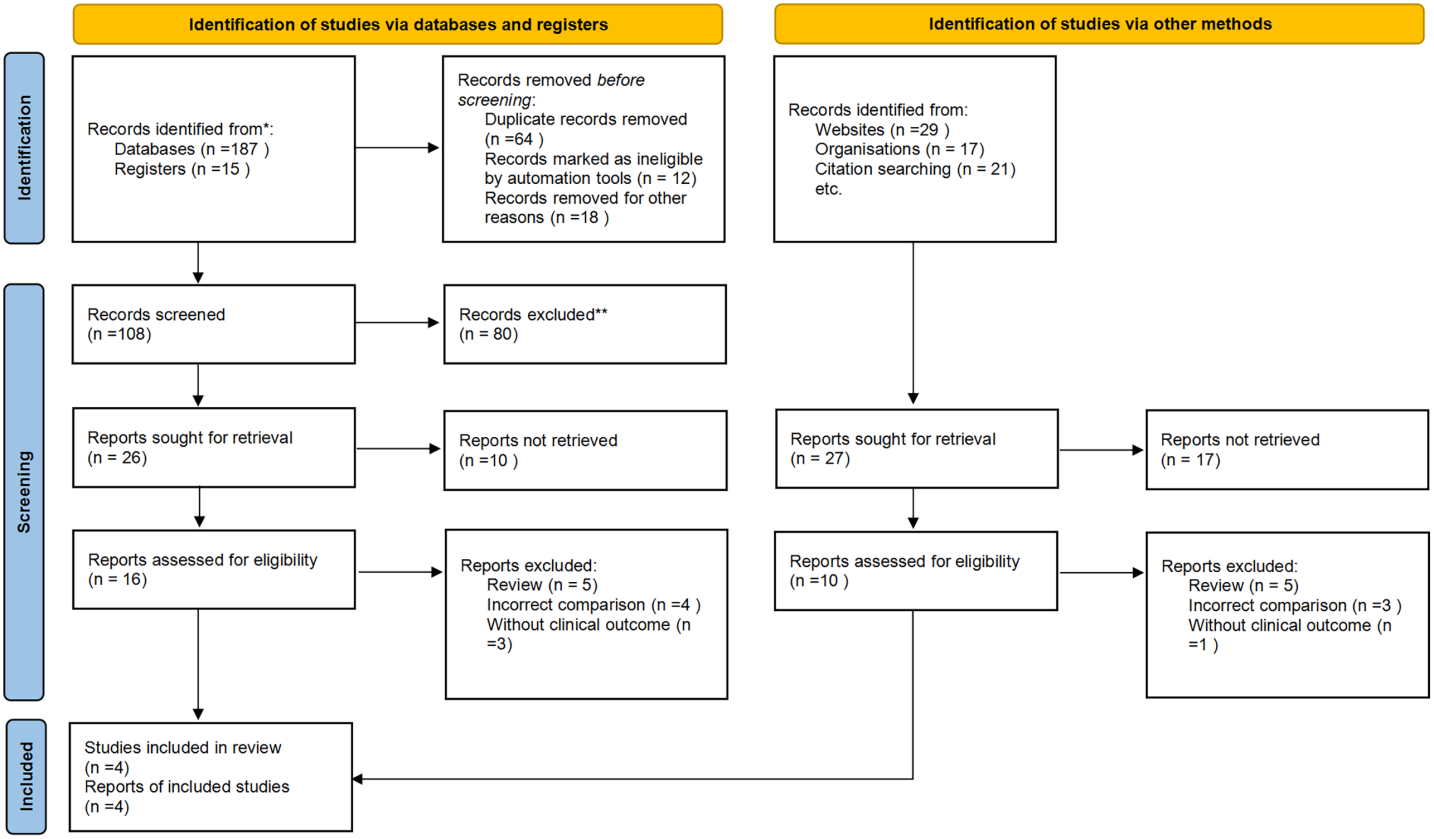
Flow diagram.

We initially identified 219 potentially relevant articles. Seventy studies were considered of interest and were retrieved for full-text review. Seventeen articles were excluded owing to duplication (n = 6), reviews (n = 5), incorrect comparisons (n = 4), and no clinical outcomes (n = 3). Therefore, four randomized trials were finally included in the analysis. **Figure 1** shows a flowchart showing the process of study selection.

### Study characteristics

Four published RCTs with a total of 1,313 patients were included, and all have completed and reported the clinical outcomes (Esposito et al., 2011; Baer et al., 2013; Dai et al., 2015; Katz et al., 2021). One RCT (Esposito et al., 2011) enrolled pediatrics with community-acquired pneumonia, two (Baer et al., 2013; Dai et al., 2015) with acute lower respiratory tract infection, and one (Katz et al., 2021) with sepsis. In all RCTs (Esposito et al., 2011; Baer et al., 2013; Dai et al., 2015; Katz et al., 2021), the antibiotic therapy regimen was modified according to the results of PCT. Antibiotic initiation was when the PCT level was >0.25 ug/L (Esposito et al., 2011; Baer et al., 2013; Dai et al., 2015; Katz et al., 2021); discontinuation was when clinical stabilization and PCT level were <0.25 ug/L (Esposito et al., 2011; Baer et al., 2013; Dai et al., 2015; Katz et al., 2021); continuation was when PCT level decreased <80% of the peak level (Dai et al., 2015); escalation was when PCT level was ≥0.5 ug/L or increased compared with peak level (Katz et al., 2021). Meanwhile, the PCT-guided antibiotic therapy regimen was overruled when life-threatening infections occurred, such as severe co-morbidity, hemodynamic, or respiratory instability (Baer et al., 2013). Meanwhile, in the empirical antibiotic regimen group, antibiotic initiation was decided based on physical examination and guidelines. Mild CAP cases took antibiotic monotherapy and severe CAP cases took beta-lactam plus macrolide therapy. The duration of antibiotic therapy was usually 7–17 days, depending on the disease severity. For uncomplicated infections, 7–10 days of antibiotic therapy is appropriate. However, for complicated and severe infections, such as sepsis, empyema, abscess, and parapneumonic effusions, 14 days or more of antibiotic therapy is required (Esposito et al., 2011; Baer et al., 2013; Dai et al., 2015; Katz et al., 2021). Primary endpoints were antibiotic prescription in three studies (Esposito et al., 2011; Baer et al., 2013; Dai et al., 2015), and antibiotic days of therapy in another study (Katz et al., 2021) (**Table 1**).

**Table 1:** Baseline characteristics of clinical trials included.

Trial/First author	Study design	Patients	PCT-guided antibiotic treatment regimen	Empirical antibiotic regimen	Study endpoint
Esposito (Esposito et al., 2011)	RCT	310 pediatrics, aged 1 month–14 years; Diagnosed as CAP; Without demonstrable complications, including pleural effusion, empyema, lung necrosis, pneumatocele	(1) initiation: PCT level was >0.25 ug/L; (2) continuation: PCT level was >0.25 ug/L; (3) discontinuation: PCT level was <0.25 ug/L;	(1) Antibiotic therapy was in accordance with the SIP guidelines; (2) Mild CAP cases took antibiotic monotherapy; (3) Severe CAP cases took beta-lactam and macrolide therapy; (4) Duration of antibiotic therapy was 7–17 days depending on disease severity	(1) Primary outcome: new antibiotic prescription; (2) Secondary outcomes: duration of fever and oxygen therapy, length of hospital stay, recurrence of respiratory symptoms, and antibiotic side effects
ProPAED (Baer et al., 2013)	RCT	337 pediatrics, aged 1month–18 years; Diagnosed as acute lower respiratory tract infection	(1) initiation: PCT level was >0.25 ug/L; (2) continuation on day 5: >1 ug/L with 7 more days; 0.51–1 ug/L with 5 more days; 0.26–0.5 ug/L with 3 more days; (3) discontinuation: clinical stabilization plus PCT level was <0.25 ug/L; (4) overruled: life threatening infection, including severe co-morbidity, emerging ICU need, hemodynamic or respiratory instability	(1) Antibiotic therapy was initiated based on physical assessment and guidelines; (2) For uncomplicated CAP, 7–10 days of antibiotic therapy is required. (3) For complicated CAP, parapneumonic effusions, empyema, abscess, 14 days or more of antibiotic therapy is needed.	(1) Primary outcome: antibiotic prescription in the first 14 days following randomization; (2) Secondary outcome: duration of antibiotic therapy, hospitalization, duration of hospital stay, antibiotic side effects, impairment of daily activity
Dai (Dai et al., 2015)	RCT	(1) 396 pediatrics, aged 1 month–18 years; (2) Diagnosed as lower respiratory tract infection	(1) initiation: PCT level was >0.25 ug/L; (2) continuation on day 5: >1 ug/L with 7 more days; 0.51–1 ug/L with 5 more days; 0.26–0.5 ug/L with 3 more days; (3) discontinuation: clinical stabilization plus PCT level was <0.25 ug/L; (4) overruled: life threatening infection	(1) Antibiotic therapy was initiated based on physical assessment and guidelines; (2) For uncomplicated CAP, 7–10 days of antibiotic therapy is required. (3) For CAP with complications, such as parapneumonic effusions, empyema, abscess, 14 days or more of antibiotic therapy is needed.	(1) Primary outcome: antibiotic prescription in the first 14 days following randomization; (2) Secondary outcome: duration of antibiotic therapy, hospitalization, duration of hospital stay, antibiotic side effects
Katz (Katz et al., 2021)	RCT	(1) 270 pediatrics, aged >7 day–18 years; (2) critically ill subjects admitted to ICU; (3) started on antibiotics within 1 day of enrollment	(1) deescalation: PCT level fell by ≥80% of peak level or was 0.25–<0.5 ug/L; (2) escalation: PCT level was ≥0.5 ug/L or increased compared with peak level; (3) continuation: PCT level decreased <80% of peak level; (3) discontinuation: PCT level was <0.25 ug/L	Received provider-directed laboratory testing and antibiotic stewardship; (2) antibiotic stewardship consisted of audit and in-person feedback (i.e., handshake stewardship) from 9 am to 5 pm Monday to Friday.	(1) Primary outcome: antibiotic days of therapy in the first 14 days following randomization; (2) Secondary outcomes: length of antibiotic therapy in the first 14 days, days of therapy of broad-spectrum antibiotic therapy, 30-day mortality, length of ICU stay, etc.

RCT, randomized clinical trial; PCT, procalcitonin; SIP, Italian Society of Pediatrics; ICU, intensive care unit; CAP, community-acquired pneumonia.

Among the pediatrics enrolled, the average age was in the range of 2.3 to 4.7 years. The total number of patients in each study was in the range of 270–337, and most were male. About 67.5% of pediatrics had pneumonia, and 14.7% had non-CAP lower respiratory tract infections. The average levels of PCT were 0.97 ug/L, C-reactive protein was 51.0 mg/L, and leukocytes were 13.7 cells/ul. Most pediatrics were followed up for less than 1 month, and the average follow-up period was 22 days (**Table 2**).

**Table 2:** Baseline characteristics of patients in the PCT-guided and empirical antibiotic therapy groups.

First author	Year	Patient’s Num.	Age, year	Male, %	Pneumonia, %	Non-CAP LRTI,%	PCT, ug/L	CRP, mg/L	leukocyte, cells/ul	Follow, d
Esposito (Esposito et al., 2011)	2011	155/155	4.3/4.7	54.8/56.8	100/100	0/0	1.8/1.8	88/71	16.3/15.2	28
ProPAED (Baer et al., 2013)	2013	168/169	2.7/2.9	58.0/58.0	64.0/63.0#	36.0/37.0	0.26/0.27	23.0/20.0	11.9/11.3	14
Dai (Dai et al., 2015)	2015	198/198	2.7/2.9	NR	63.1/62.1	26.9/27.9	0.25/0.21	22/20	11.8/11.4	14
Katz (Katz et al., 2021)	2021	137/133	2.3/1.6	58.4/45.1	37.2/40.6$	8.8/4.5&	0.9/0.8	NR	NR	28

#, community-acquired pneumonia; $, pneumonia plus aspiration pneumonia; &, tracheitis; PCT, procalcitonin; Num., number; CAD, community-acquired pneumonia; LRTI, lower respiratory tract infection; CRP, C-reactive protein; d., day; NR, not reported.

### Methodological quality assessment

**Figure 2:**
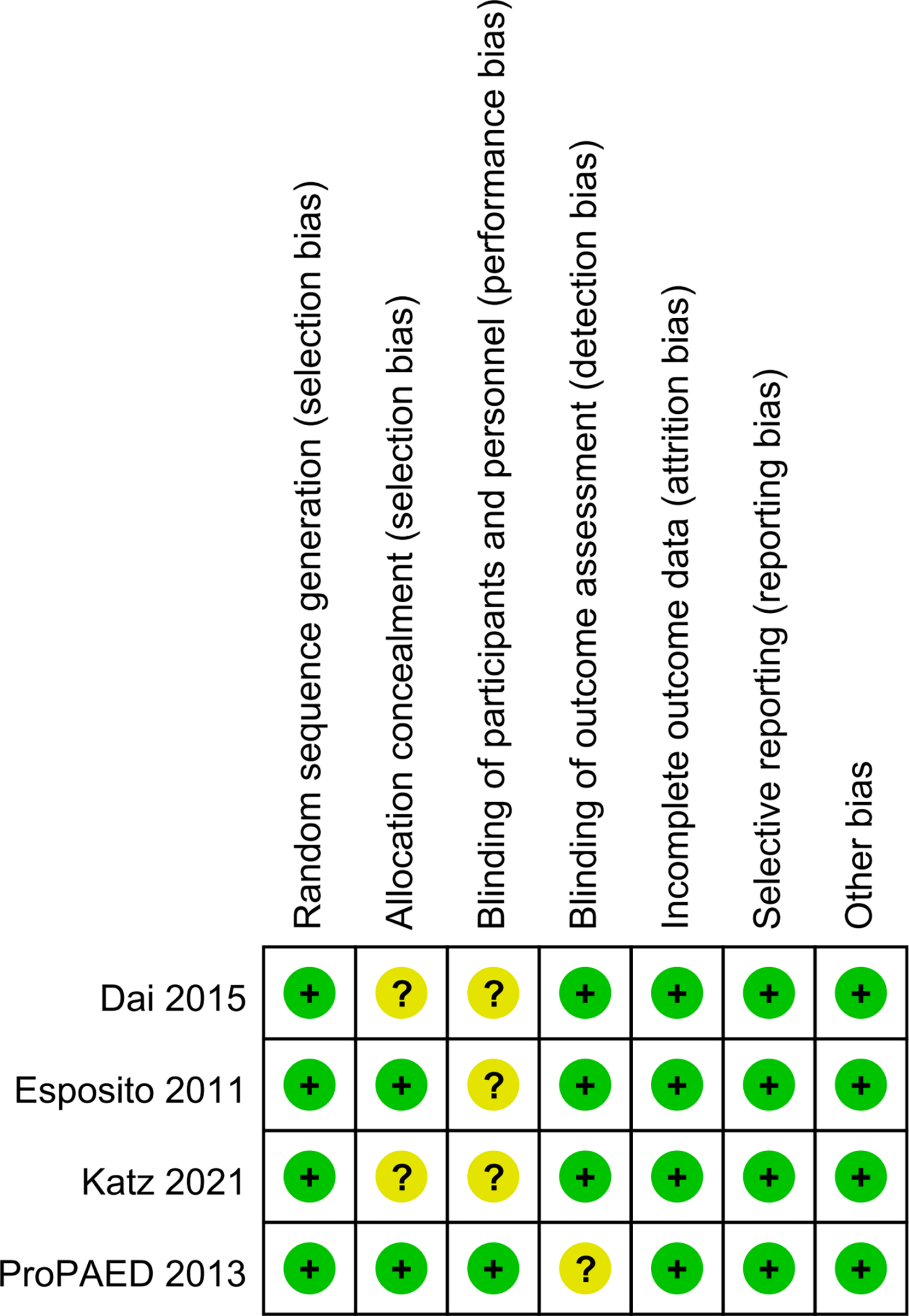
Quality evaluation with Cochrane’s risk of bias tool.

Four trials randomized the participants and reported the details about the means of generation of random sequences (Esposito et al., 2011; Baer et al., 2013; Dai et al., 2015; Katz et al., 2021). Two studies used satisfactory methods of concealed treatment allocation (Baer et al., 2013; Dai et al., 2015). Blinding of participants and personnel was reported in two studies (Baer et al., 2013; Dai et al., 2015). There was a low risk of attrition bias and reporting bias in all studies (**Figure 2**).

### Primary endpoint

**Figure 3:**
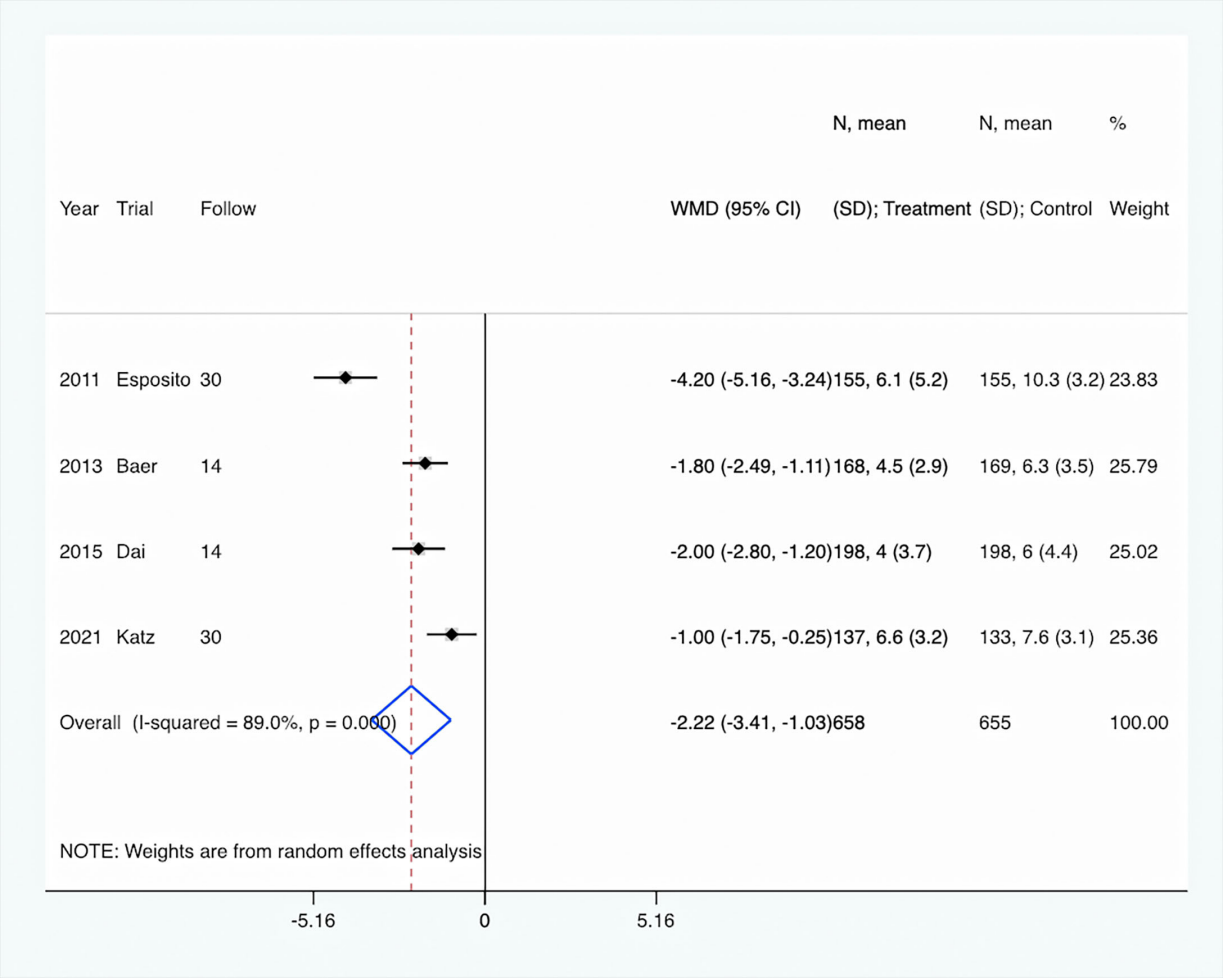
PCT-guided treatment was associated with shorter length of antibiotic therapy.

The length of antibiotic therapy: All four trials (Esposito et al., 2011; Baer et al., 2013; Dai et al., 2015; Katz et al., 2021) provided data about the length of antibiotic therapy. There were 658 pediatrics in the PCT-guided antibiotic therapy group and 655 patients in the control group. Compared to the control group, PCT-guided antibiotic therapy was associated with a significantly shorter length of antibiotic therapy of about 2.22 days (WMD, −2.22 days; 95% CI, −3.41 to −1.03; P <0.001) (**Figure 3**). There was a high level of heterogeneity (I^2^ = 89.0%). However, sensitivity analysis was performed by removing each of the trials individually, which did not detect any influence on the length of antibiotic therapy.

### Secondary endpoints

**Figure 4:**
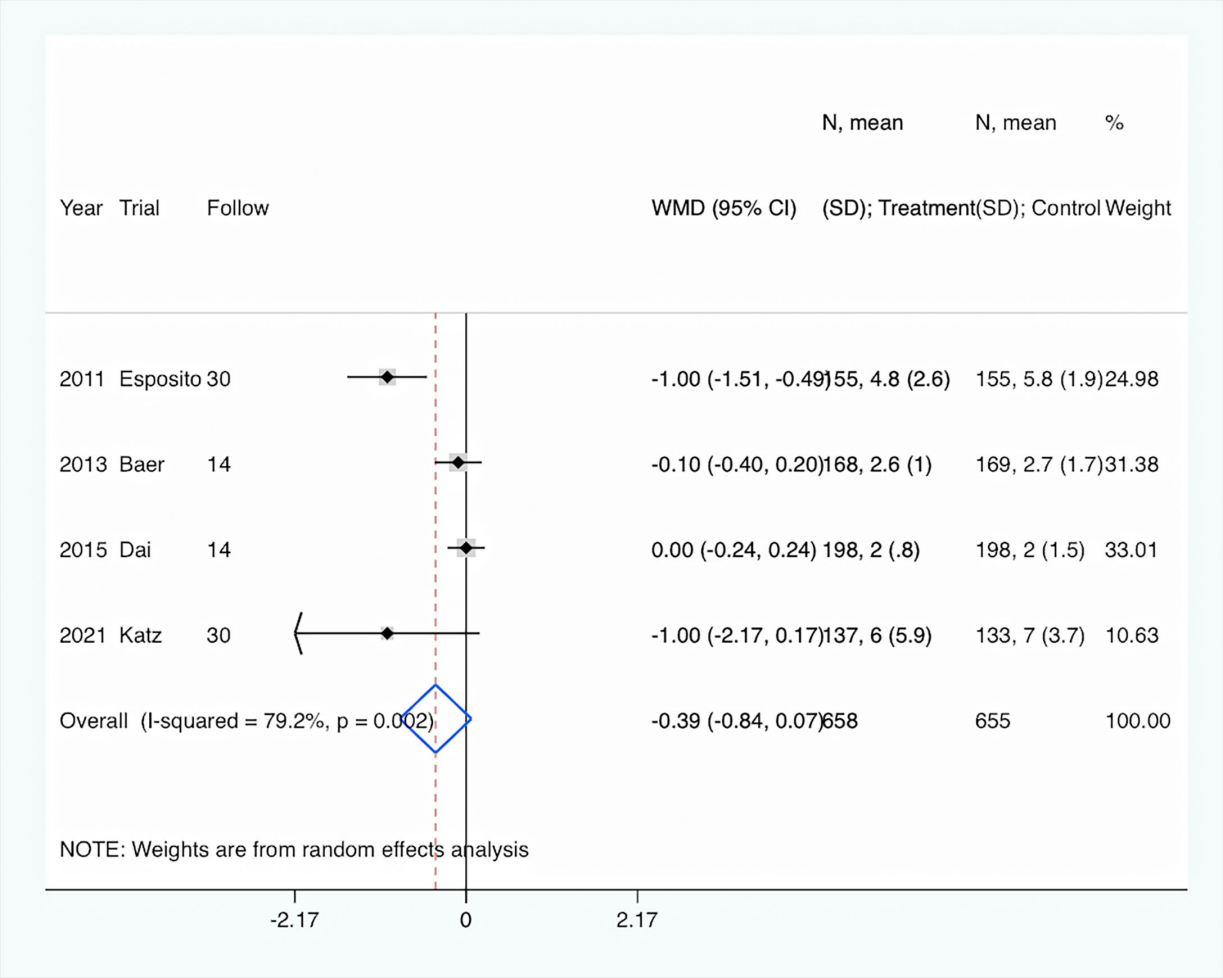
PCT-guided treatment was associated with similar length of hospital stay.

Length of hospital stay: Length of hospital stay was shown in all four trials (Esposito et al., 2011; Baer et al., 2013; Dai et al., 2015; Katz et al., 2021). Overall, PCT-guided antibiotic therapy was associated with a comparable length of hospital stay compared to the control group (WMD, −0.39 days; 95% CI, −0.84 to 0.07; P = 0.094) (**Figure 4**). In addition, there was a high level of heterogeneity (I^2^ = 79.2%).

**Figure 5:**
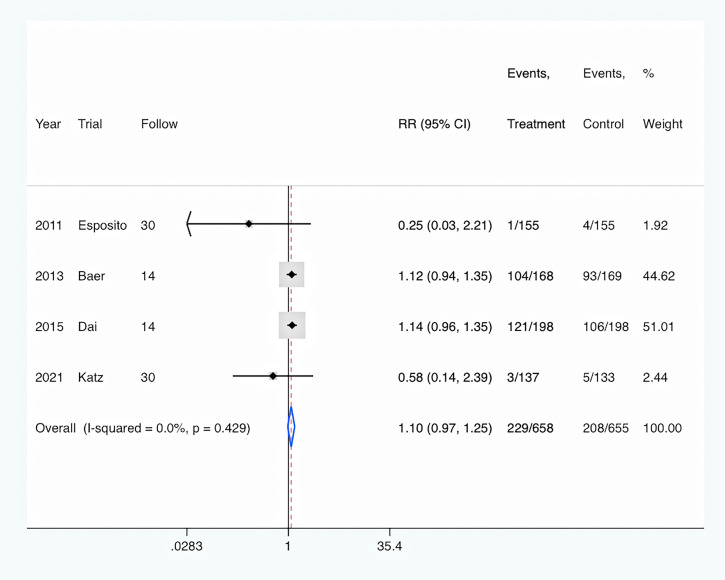
PCT-guided treatment was associated with similar rate of antibiotics prescription.

Antibiotics prescription: The rate of antibiotic prescription was shown in four trials (Esposito et al., 2011; Baer et al., 2013; Dai et al., 2015; Katz et al., 2021). Overall, PCT-guided antibiotic therapy was associated with a similar rate of new antibiotic prescription compared to the control group (RR, 1.10; 95% CI, 0.97–1.25; P = 0.122) (**Figure 5**). And there was a low level of heterogeneity (I^2^ = 0).

**Figure 6:**
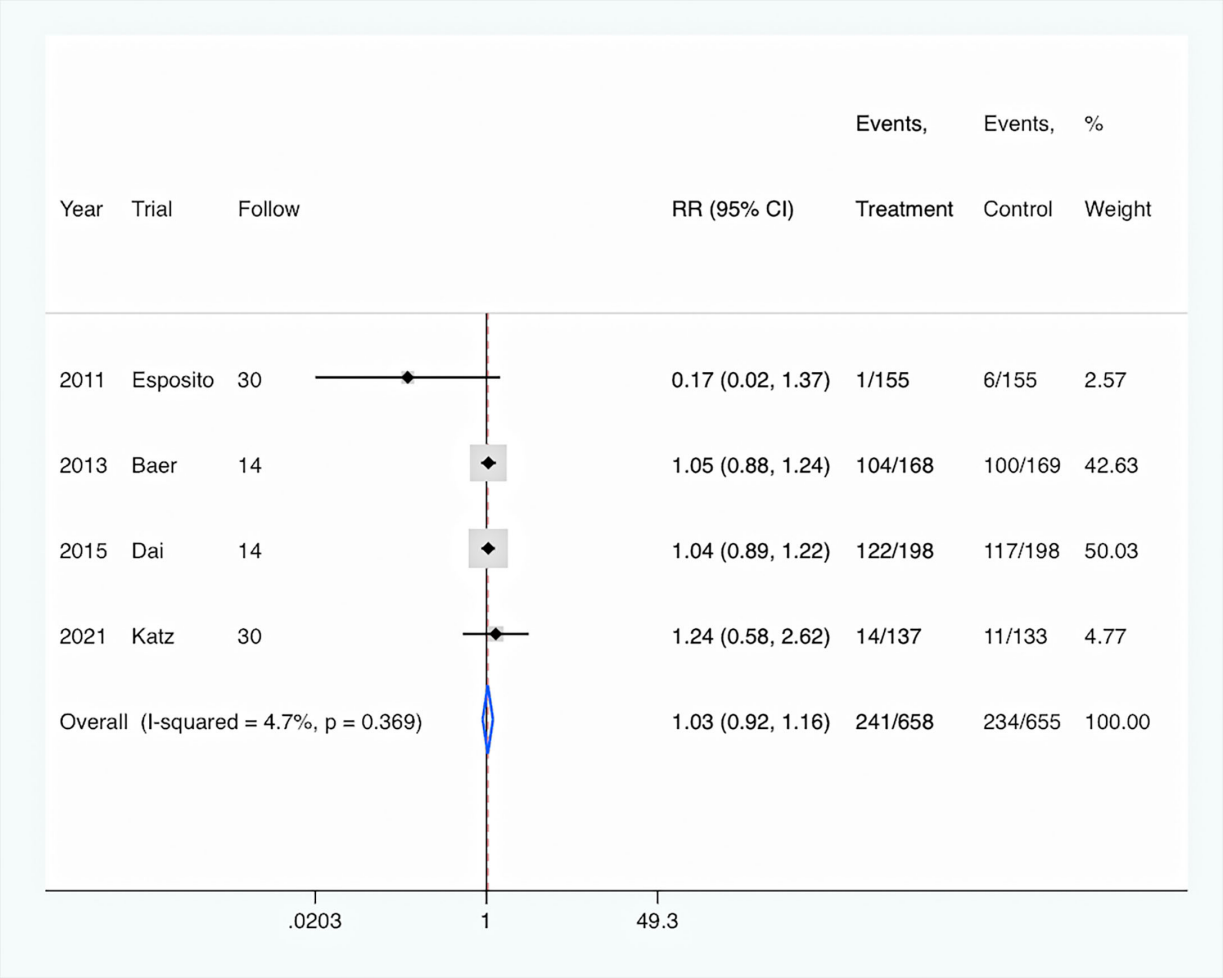
PCT-guided treatment was associated with similar rate of hospital readmission.

Hospital readmission: All four studies (Esposito et al., 2011; Baer et al., 2013; Dai et al., 2015; Katz et al., 2021) reported the rates of hospital readmission. Overall, PCT-guided antibiotic therapy was not associated with significantly decreased rates of hospital readmission (RR, 1.03; 95% CI, 0.92–1.16; P = 0.613) compared with the control group (**Figure 6**). Meanwhile, there was a low level of heterogeneity (I^2^ = 4.7%).

Mortality: Only one study (Katz et al., 2021) reported the rates of mortality. Overall, PCT-guided antibiotic therapy was associated with similar rates of mortality (RR, 0.73; 95% CI, 0.17–3.19; P = 0.674) compared with the control group.

**Figure 7:**
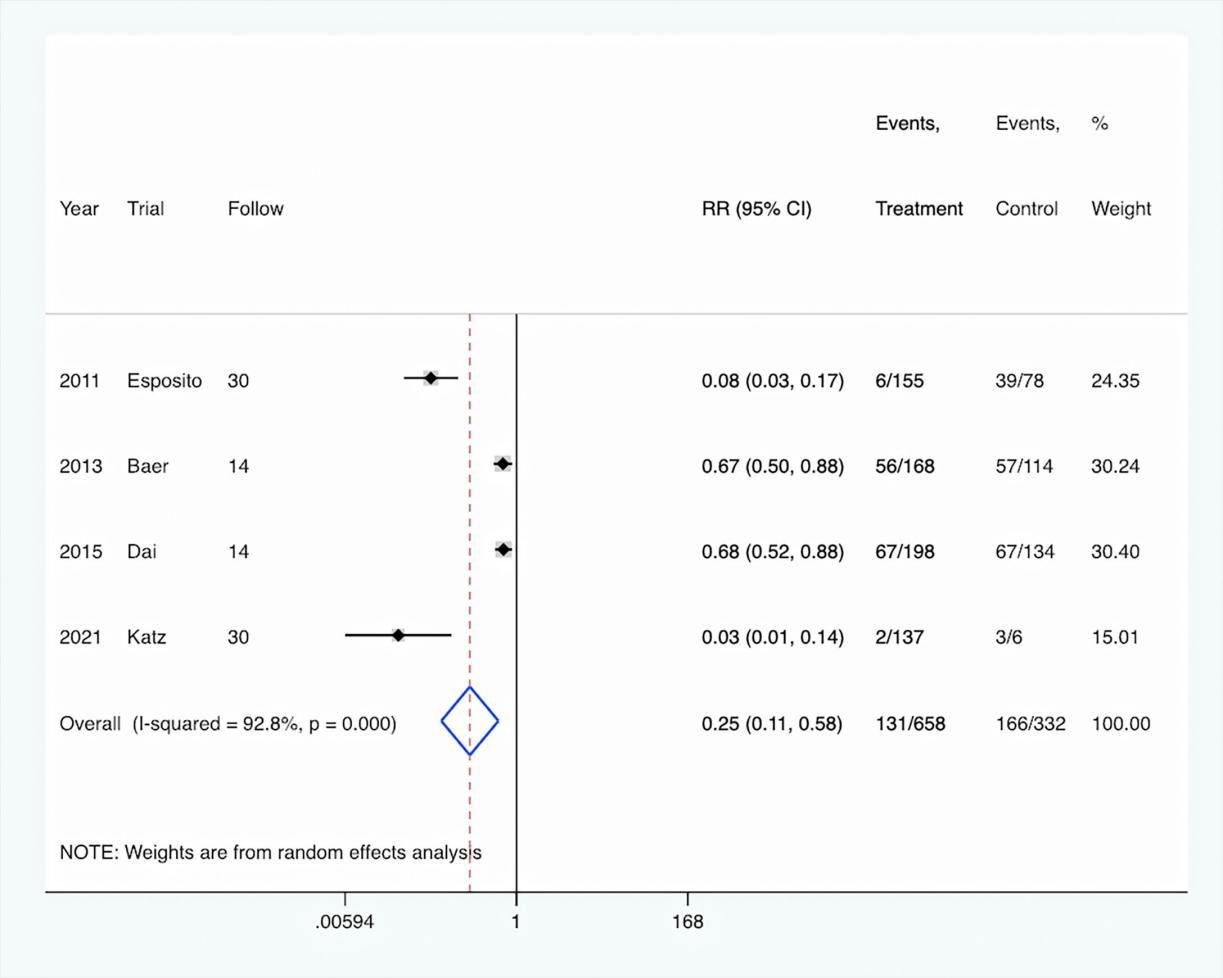
PCT-guided treatment was associated with decreased rate of antibiotic adverse event.

Antibiotic adverse event: The rate of antibiotic adverse events was reported in four studies (Esposito et al., 2011; Baer et al., 2013; Dai et al., 2015; Katz et al., 2021), and was significantly decreased in the PCT-guided antibiotic therapy group compared with the control group (RR, 0.25; 95% CI, 0.11–0.58; P <0.001) (**Figure 7**). Meanwhile, there was a high level of heterogeneity (I^2^ = 92.8%).

### Trial sequential analysis

**Figure 8:**
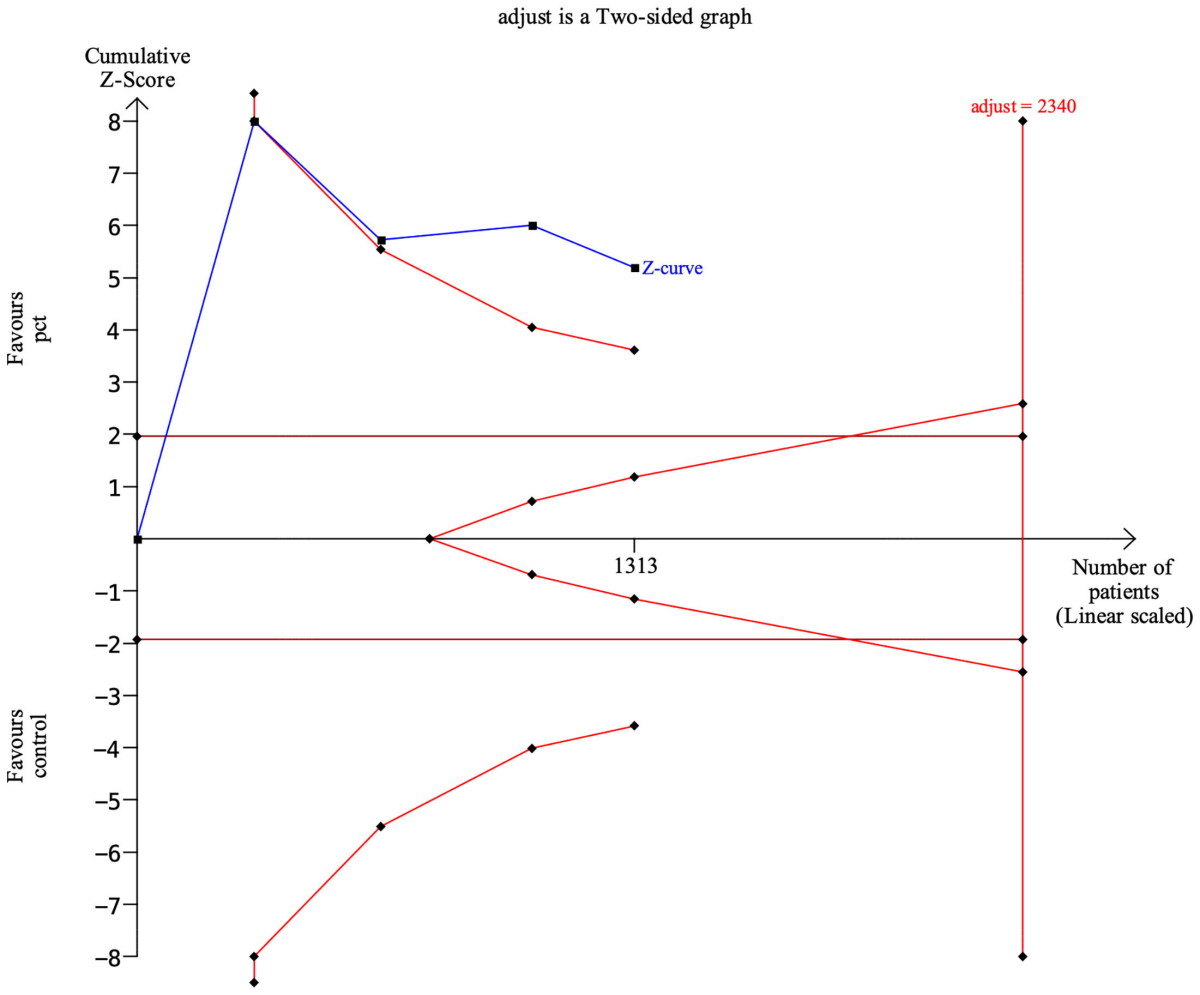
Trial sequential analysis.

The TSA analysis showed that, assuming a mean difference of 2.0 days between PCT-guided antibiotic therapy and control groups in the length of antibiotic therapy, the RIS required 2,340 participants. The cumulative Z curve crossed trial sequential boundaries, indicating statistically significant differences in the length of antibiotic therapy between groups (**Figure 8**).

## Discussion

This meta-analysis, including all the available evidence, showed that the application of PCT can guide the antibiotic management of pediatrics with infective disease, not only shorten the length of antibiotic therapy (WMD, −2.22 days; 95% CI, −3.41 to −1.03), but also decrease the risk of antibiotic adverse events (RR, 0.25; 95% CI, 0.11–0.58).

In addition to assessing and monitoring the severity of disease, PCT can also be used to guide antibiotic therapy. The SAPS studies (de Jong et al., 2016) included adult patients with suspected or confirmed bacterial infection in the ICU, and evaluated the effect of a PCT level-guided strategy on antibiotic exposure time and mortality. In the study, 49% were hospital-acquired infections and 64% were lung infections. The PCT-guided group discontinued antibiotics when PCT was <0.5 ug/L or decreased by ≥80% (de Jong et al., 2016). The results showed that compared with the control group, the exposure time of antibiotics in the PCT-guided group was markedly shortened (7.5 day *vs*. 9.3 day). Meanwhile, 28-day (20% *vs*. 25%) and 1-year mortality (36% *vs*. 43%) were significantly lower in the PCT-guided group (de Jong et al., 2016). In the ProVAP study, for enrolled patients with ventilator-associated pneumonia, after 72 h of initial antibiotic treatment, the reduction or discontinuation of antibiotic dose is adjusted according to the PCT level (Stolz et al., 2009): (1) When PCT is <0.25 ug/L, it is strongly recommended not to use antibiotics; (2) PCT is at 0.25–0.5 ug/L or decrease by ≥80%, it is recommended to reduce or stop antibiotics; (3) PCT is ≥0.5 ug/L or decrease by <80%, which indicates that bacterial infection still exists, and dose reduction or discontinuation is not recommended; (4) When PCT is >1 ug/L, discontinuation is strongly not recommended. The authors found that compared with the control group treated according to the guideline, the overall duration of antibiotic therapy in the PCT-guided group was significantly shortened by 27% (P = 0.038). Moreover, after 72 h of initial treatment, the proportion of patients who were de-escalated to monotherapy in the PCT-guided group was significantly higher than the control group (54% *vs*. 28.6%, P = 0.008) (Stolz et al., 2009). Recently, the PROGRESS study confirmed that in inpatients with sepsis, PCT-guided antibiotic therapy was more effective in reducing infection-associated adverse events (7.2% *vs*. 15.3%), 28-day mortality (15.2% *vs*. 28.2%), length of antibiotic therapy (5 days *vs*. 10 days) and cost of hospitalization (P <0.001) than the standard of care guided by guidelines (Kyriazopoulou et al., 2021). Among 1,656 patients who enrolled in the largest RCT multicenter trial to date, the ProACT trial, which randomized patients to PCT-guided or usual care groups in 14 US emergency departments, there was no statistically significant difference between PCT and usual care in terms of antibiotic therapy duration or adverse outcomes (Huang et al., 2018; Huang et al., 2020). However, in the ProACT study, algorithm adherence varied significantly and was 39% for those with CAP. In addition, nearly half of published RCTs showed no significant reductions in antibiotic exposure with PCT-guided management (Briel et al., 2008; Schuetz et al., 2009; Huang et al., 2018; Daubin et al., 2018; Wussler et al., 2019; Huang et al., 2020), which may be associated with new progress in antibiotic stewardship or access to infectious disease diagnostics in the empirical group according to clinical guidelines. In a prospective twin-center cohort study, Lee et al. found that combining point-of-care viral tests with PCT levels could reduce hospital stays, intravenous antibiotics, and antibiotic escalation. The combination of PCT and Rapid Multiplex Respiratory Virus Testing could therefore be another factor to evaluate the effects of PCT-guided antibiotic use (Lee et al., 2020; Su et al., 2022). In a patient-level meta-analysis, Schuetz et al. enrolled 6,708 adult patients from 26 RCTs. Similar to our study, they found that PCT-guided antibiotic treatment for acute respiratory infections was associated with significantly lower mortality at 30 days (aOR = 0.83, 95%CI: 0.70–0.99), a 2.4-day reduction in antibiotic exposure (5.7 *vs*. 8.1 days) and a reduction in antibiotic-related side-effects (aOR = 0.68, 95% CI = 0.57–0.82) (Schuetz et al., 2018). Among those with acute exacerbations of chronic obstructive pulmonary disease (AECOPD), Mathioudakis et al. included 1,062 patients from eight trials and confirmed that PCT-based protocols decreased antibiotic prescription (RR = 0.56, 95% CI: 0.43–0.73) and antibiotic exposure (MD = −3.83 days, 95% CI: −4.32 to −3.35 days). However, all these studies enrolled adult patients (Mathioudakis et al., 2017). Therefore, it is difficult to extrapolate from these RCTs to the use of a PCT-guided algorithm in children with infectious disease.

In contrast to the numerous published trials enrolling adult patients, there are only four pediatric (Esposito et al., 2011; Baer et al., 2013; Dai et al., 2015; Katz et al., 2021) and two neonatal (Lee et al., 2020; Su et al., 2022) RCTs. The ProPAED trial enrolled 337 subjects with LRTIs and divided them randomly into two groups: the PCT-guided antibiotic management group (n = 168) and the usual care group (n = 169). The antibiotic management days were significantly fewer in the PCT-guided group compared with the control group (4.5 days *vs*. 6.3 days) (Baer et al., 2013). In another study, Esposito and his colleagues enrolled 310 hospitalized children with uncomplicated CAP and randomized them into the PCT-based management group (n = 155) or usual care group (n = 155). After follow-up, they found that the PCT-based group was associated with a significantly shorter antibiotic duration (5.37 days *vs*. 10.96 days). In another study of 396 pediatrics with LRTIs, there were 198 subjects in each group. Dai et al. found that the length of antibiotic therapy was significantly decreased in the PCT-guided group (4 days *vs*. 6 days). Meanwhile, in neonates with sepsis, PCT-guided therapy was also associated with markedly reduced duration of antibiotic therapy (P <0.001) (Stocker et al., 2010; Stocker et al., 2017). However, Katz et al. recently conducted a RCT that enrolled 270 pediatrics with sepsis, and randomized them into the PCT-guided management group (n = 137) and usual care group (n = 133). The authors found that antibiotic days of therapy were not markedly different between the two groups (6.6 days *vs*. 7.6 days). Similar to previous studies, our study confirmed that PCT-guided antibiotic therapy was associated with a significantly shorter length of antibiotic therapy of about 2.22 days compared to the control group (WMD, −2.22 days; 95% CI, −3.41 to −1.03; P <0.001).

Clinical guidelines for antibiotic management vary in their recommendations on PCT-guided management for pediatrics with pneumonia, reflecting the conflicting evidence in this area of research (Schuetz et al., 2019). Our study aimed to remove this uncertainty by recruiting more participants to detect a significant difference in antibiotic exposure. Of relevance, the point estimate for the length of antibiotic therapy reported showed the intervention to be more effective (WMD, −2.22 days; 95% CI, −3.41 to −1.03) compared with empirical management. A sample size calculation indicates that the TSA analysis showed that, assuming a 20% difference between the two groups, the RIS required 2,340 participants, and the accrued information size was 1,313. Therefore, more large-scaled studies are needed for even pooled evidence to have sufficient power.

Nevertheless, the evidence for this effect is not yet robust. In a recently published study, Sekmen et al. included 488 children (<18 years) with pneumonia. PCT was tested at enrollment (median, 0.37 ng/ml; IQR, 0.11 to 2.38 ng/ml). They found that PCT values were not significantly associated with higher rates of antibiotic initiation (OR = 1.02; 95% CI, 0.97%–1.06%) or empirical antibiotic selection (OR = 1.07; 95% CI, 0.97%–1.17%) (Sekmen et al., 2022). Therefore, other factors may influence antibiotic therapy decisions. Meanwhile, among children and young patients with cancer, febrile neutropenia is a common complication. Morgan et al. conducted a single-arm pilot study and showed a reduction in antibiotic duration or spectrum among 4/8 (50%) of episodes without clear microbiologically documented or clinical infection (Morgan and Phillips, 2022). Thus, there is still not sufficient evidence to conclude definitively on the effectiveness of PCT-guided treatment in the management of pediatric with infective disease, and particularly in more severely pneumonia, sepsis patients or febrile neutropenia, such as those managed in emergency center (Velissaris et al., 2021). Reporting of patient and study characteristics needs to be broader and more detailed to allow further exploration of study populations. Many PCT-guided intervention strategies evaluated in this meta-analysis are multifaceted. All previous trials enrolled pediatrics with different infectious disease (tracheitis, pneumonia, sepsis), had low adherence to the algorithm, and had small sample sizes. Furthermore, the effect of PCT-guided management in pediatrics with severe CAP or those with high compliance with the algorithm was not evaluated. Therefore, the applicability of PCT-guided antibiotic management for pediatrics to current practice needs to be confirmed in more studies.

### Limitations

There are several limitations. (1) Our meta-analyses are based on study-level data with the flaws of the original study. (2) There is a risk of geographical variations. All four studies showed slight differences in the characteristics of the patient, conditions, PCT-guided treatment strategies, and following period. (3) The sample size is small. The TSA analysis showed that, assuming a 20% difference between groups in the length of antibiotic therapy, the RIS required 2,340 participants, and the accrued information size was 1,313.

## Conclusions

On the basis of our pooled analysis, PCT-guided therapy appears to reduce antibiotic therapy duration and adverse event risks in pediatric patients with infectious diseases. However, the sample size is still small, and there is insufficient evidence to reach a definitive conclusion on the final effectiveness.

## Data availability statement

The raw data supporting the conclusions of this article will be made available by the authors, without undue reservation.

## Ethics statement

Ethical review and approval was not required for the study on human participants in accordance with the local legislation and institutional requirements. Written informed consent from the participants’ legal guardian/next of kin was not required to participate in this study in accordance with the national legislation and the institutional requirements.

## Author contributions

PL wrote the main manuscript text. PL and JiaL analyzed the data. JiaL designed this work. All authors contributed to the article and approved the submitted version.

## Conflict of interest

The authors declare that the research was conducted in the absence of any commercial or financial relationships that could be construed as a potential conflict of interest.

## Publisher’s note

All claims expressed in this article are solely those of the authors and do not necessarily represent those of their affiliated organizations, or those of the publisher, the editors and the reviewers. Any product that may be evaluated in this article, or claim that may be made by its manufacturer, is not guaranteed or endorsed by the publisher.
